# Barriers Associated with Help-Seeking for Stroke Symptoms Despite Public Awareness Campaigns: A Cross-Sectional Study

**DOI:** 10.3390/neurosci7030070

**Published:** 2026-06-14

**Authors:** Sheharyar S. Baig, Mudasar Aziz, Sara Sara, Sarah Ingram, Arshad Majid, Elizabeth Abbey, Lucy A. Eaves, Noor Sharrack, Ali Ali, Jessica N. Redgrave

**Affiliations:** 1 Sheffield Institute of Translational Neuroscience, Department of Neuroscience, University of Sheffield, 385A Glossop Road, Sheffield S10 2HQ, UK; sbaig1@sheffield.ac.uk (S.S.B.); mudasar.aziz@nhs.net (M.A.);; 2 North Lincolnshire and Goole Foundation Trust, Woodland Avenue, Goole DN14 6RX, UK; 3 Royal Cornwall Hospitals Trust, Treliske, Truro, Cornwall TR1 3LJ, UK; sarah.ingram6@nhs.net; 4 Sheffield Teaching Hospitals NHS Foundation Trust, Glossop Road, Sheffield S10 2JF, UK; 5 Sheffield Health Partnership University NHS Foundation Trust, Centre Court, Atlas Way, Sheffield S4 7QQ, UK

**Keywords:** stroke, thrombolysis, emergency department utilisation

## Abstract

Background: The nationally advertised mass media campaign Act-FAST UK, delivered in multiple waves since its launch in 2009, has increased public awareness of stroke symptoms. However, many stroke patients still delay in calling for help and reach the hospital too late to receive emergency treatments. The reasons for this cognitive dissonance between recognition of symptoms and urgent seeking of emergency medical services (EMS) are unclear. Aims: This study aimed to quantify cognitive, psychological, and knowledge-based barriers to help-seeking in patients with acute stroke or transient ischaemic attack (TIA), as well as in intervening witnesses, and to examine their association with the use of EMS as the initial point of contact. Methods: We interviewed patients admitted to a hyperacute stroke unit with a stroke or transient ischaemic attack (TIA) from 2013 to 2016. People who contacted emergency services on the patient’s behalf (intervening witnesses (IWs)) were also interviewed when available. Reasons given for delays in calling for help were related to correct symptom recognition, and whether/at what time, emergency services were contacted after symptoms onset. Results: A total of 602 patients (429 with stroke, 173 with TIA) along with 128 witnesses who intervened in calling for help in those cases (IWs) were interviewed. In the subset of patients with both measures available, there was a strong positive correlation between NIHSS score and number of FAST symptoms (Spearman’s rho = 0.645, *p* < 0.001), providing supportive evidence for the use of FAST symptom count as a proxy measure of stroke severity. A total of 469 (77.9%) of the patients were aware of a media education campaign about stroke, but only 145 (24.1%) had attributed their own symptoms to stroke at onset. However, correct self-diagnosis of stroke was not associated with direct calls to the EMS (OR 1.43, 95% CI 0.84–2.45). Cognitive, psychological or emotional barriers to help-seeking, as reported by prior published studies, were reported by 463 (81.2%) of the patients we interviewed but in only 63 (53.3%) of the IWs (*p* < 0.001). Amongst the patient cohort, “not thinking symptoms were serious” (275, 45.7%) and “waiting to see if symptoms would go away” (285, 47.3%) were most strongly negatively associated with EMS use (OR 0.52, 95% CI 0.32–0.84 and OR 0.34, 95% CI 0.21–0.55, respectively). Only 55 (9.1%) of the patients interviewed had been aware of any time-critical stroke treatment prior to their stroke. Eighteen stroke patients (4.2%) reached hospital in time to receive thrombolysis, but an additional 170 (39%) could have been considered for this treatment (i.e., had no apparent other contraindications from a notes review) had they arrived within 4 h of symptom onset. Conclusions: Future public education campaigns may be more effective if they specifically address factors associated with delays in calling for help after stroke symptoms and emphasise the existence of emergency treatments, which are also time-critical. More effective public education may have the potential to increase the proportion of patients arriving in time to benefit from such treatments.

## 1. Introduction

Stroke is a medical emergency with time-dependent treatments, including reperfusion therapies for ischaemic stroke and urgent interventions for haemorrhagic stroke. Thrombolysis uses medication to dissolve blood clots, while thrombectomy is a minimally invasive procedure that physically removes clots to restore cerebral blood flow. When delivered promptly, typically within 4.5 h of symptom onset for thrombolysis and within 24 h for thrombectomy, these interventions significantly reduce mortality and disability [1]. However, their effectiveness depends on rapid symptom recognition by patients, relatives and first responders, and timely presentation to medical services.

National campaigns such as Act-FAST (Face–Arm–Speech–Time) have sought to enhance the early recognition of stroke symptoms amongst the general public and increase timely use of emergency services. In the UK, there has been increased utilisation of emergency medical services (EMS; ambulance call-out or self-referral to the emergency department) for acute stroke following each wave of the Act-FAST campaign [2,3] since its launch in 2009. However, the median time from stroke onset to arrival at a hospital has increased over the last decade [4].

Although the 2019 NHS England Long-Term Plan set a target for 10% [5] of stroke patients to receive thrombectomy, the current delivery rate remains around 3% [6,7]. This shortfall is due, in part, to lack of 24/7 provision of services in all centres [5,6,7]. However, even where services are available, patients often arrive beyond the early time window for treatment and require advanced imaging to determine whether a salvageable brain remains. In all eligible cases, the sooner the thrombectomy is performed, the better the chance of a good outcome for the patient [8].

In the case of thrombolysis, while NHS England targets a 20% thrombolysis rate, current national practice achieves only 11–12% of all stroke patients [9]. The most common reason for ineligibility is arrival at hospital outside of the therapeutic time window. Delays to hospital arrival may be associated with poor recognition of stroke symptoms by the patient or by those people in the vicinity of the patient, or a lack of awareness of the need for urgency [10]. Reducing delays in contacting emergency medical services has been identified as a key priority for public education.

Several qualitative studies, in stroke patients and in healthy populations at risk of stroke (e.g., elderly cohorts), have elucidated a range of cognitive and emotional barriers to seeking emergency help [10,11]. These include not realising symptoms were serious, attribution to an alternative more benign cause, or not wanting to be a burden on the system. However, the relative prevalence of these barriers in (a) patients with recent stroke symptoms and (b) those who intervened in calling for help on their behalf (intervening witnesses, IWs), and their respective influence on help-seeking behaviour at the time of the episode, are poorly understood. This study aimed to quantify cognitive, psychological, and knowledge-based barriers to help-seeking in patients with acute stroke or transient ischaemic attack (TIA), as well as in intervening witnesses, and to examine their association with the use of EMS as the initial point of contact.

## 2. Methods

### 2.1. Study Design and Setting

We conducted a cross-sectional study at the hyperacute stroke unit at Royal Hallamshire Hospital, Sheffield, UK. The HASU serves approximately 500,000 people as the sole admitting centre for hyperacute stroke and TIA. Data collection occurred during four periods between August 2013 and October 2016 (August 2013, April–August 2014, January–March 2015, and June–October 2016). The researchers conducted the interviews between 9 and 5 pm Monday–Friday. Data collection was conducted in discrete periods due to researcher availability and logistical constraints, rather than continuous recruitment; however, there was no systematic selection of participants beyond these practical limitations.

### 2.2. Participants

Patients were eligible if diagnosed with acute stroke (ischaemic or haemorrhagic) or TIA according to AHA/ASA definitions [12,13]. For patients lacking capacity, relatives were approached for assent and, if granted, they answered questions on the patient’s behalf. TIA patients were predominantly interviewed immediately following TIA clinic attendance, although some were inpatients who had presented with ongoing symptoms that were resolved whilst in hospital.

If another person was responsible for calling the EMS (e.g., if the patient was incapacitated or had refused to call) and where that person (henceforth referred to as an intervening witness, IW) was present with the patient at the time of consent for the study, they were also invited to participate and were interviewed with a shortened version of the questionnaire to determine any barriers to help-seeking.

### 2.3. Data Collection

All interviews used standardised questionnaires (Supplementary File 1) and were conducted face-to-face within 72 h of admission, either at the bedside or in a private room. Participants with aphasia were included if they had capacity to give consent or if a relative could provide assent. They received additional time and communication aids as needed.

Questionnaires were based on the Response to Symptoms Questionnaire [14] and Oxford Vascular Study questionnaire [15]. Baseline characteristics were collected, including age, sex, postcode, and highest education level (basic education, higher education aged >16, or degree level). Socioeconomic status was derived from home postcodes using the Index of Multiple Deprivation (IMD) tool [16], expressed in UK quintiles (1 = least deprived, 5 = most deprived). Baseline characteristics of patients and intervening witnesses are presented in Table 1.

The nature and timing of presenting symptoms and subsequent calls for help, including who called for help and who they contacted first (EMS, GP/primary care doctor, or NHS 111 helpline), were documented.

Where available, National Institutes of Health Stroke Scale (NIHSS) scores [17] were recorded from admission notes. Patients with NIHSS ≥ 5 were considered to have at least moderate stroke severity. There were some missing data for NIHSS scores for the stroke patients, so a proxy marker of stroke severity (number of FAST symptoms) was also collected. This proxy was used pragmatically and has not been formally validated as a standalone measure; however, in patients with both measures available there was a moderate-to-strong positive correlation between FAST symptom count and NIHSS score (Spearman’s rho = 0.645, *p* < 0.001; *n* = 294). Hospital and ambulance records were viewed to corroborate participant information, including the precise timing of initial EMS calls.

We recorded whether patients received thrombolysis. For those arriving beyond the time window to allow thrombolysis within 4.5 h (assuming a door-to-needle time of 30 min), a consultant stroke physician (J.N.R.) reviewed the patient’s notes to determine their potential for eligibility had they arrived sooner. For wake-up strokes, the time when the patient or IW first became aware of symptoms was taken as the onset time for calculating the delay to help-seeking. Wake-up strokes were not considered eligible for thrombolysis in accordance with the local best-practice guidelines that were in place at the time of the study.

### 2.4. Knowledge and Barrier Assessment

Participants were asked what they initially thought had caused their symptoms and whether they were aware of any stroke media campaigns. They were asked what “FAST” stood for (correct answers: facial weakness, arm weakness, speech disturbance, time to call 999) and whether they knew of any time-dependent stroke treatments and, if so, to describe them.

Participants selected from a list of frequently reported barriers to help-seeking [18,19] (Table 2) from previously published literature, and were asked to share any additional barriers they felt had contributed to any reticence they might have felt when calling EMS.

### 2.5. Statistical Analysis

Prevalence of barriers to help-seeking was reported separately for the patients and the IWs, stratified by whether participants had correctly identified the symptoms as being due to stroke. We related demographic, clinical, knowledge-based factors, and psychological or emotional barriers to help-seeking to decisions to call EMS. Student’s independent samples *t*-test was used for continuous variables and Pearson’s χ^2^ test for categorical variables. Multivariate analysis (adjusting for age, sex, and number of FAST symptoms) was used to determine independent associations, with covariates selected a priori based on clinical relevance (age, sex, and stroke severity, approximated by number of FAST symptoms).

Missing data are reflected in the denominators used to calculate percentages. Substantial missing data are highlighted in the text and table legends. *p* < 0.05 was considered statistically significant. Analyses were performed using SPSS (IBM SPSS Statistics for Windows, V22.0).

### 2.6. Ethics and Patient Involvement

All participants provided informed consent before interviews. The study was approved by the local Ethics Committee (13/EM/0051). A community group of young stroke survivors and carers assisted in developing the questionnaire and participant information leaflets.

## 3. Results

### 3.1. Clinical Characteristics

We interviewed 602 patients (429 stroke, 173 TIA) and 128 IWs, i.e., 730 participants altogether.

In the stroke/TIA patient cohort, the mean (SD) patient age was 71.8 (14.2) years, and 325 (54.0%) were male. Median (IQR) NIHSS score at admission was 3 (1–6), range 0–27 (available for 317 (52.7%) patients). The number of FAST-positive symptoms (0, 1, 2, or 3) was used as a stroke severity marker in the multivariate model due to missing NIHSS data. For 294 cases with both measures available, there was strong positive correlation between NIHSS score and FAST symptoms (Spearman’s rho = 0.645, *p* < 0.001).

The 128 IWs included spouses, friends, and relatives. Mean (SD) age was 64.7 (16.9) years, and the IWs were predominantly female (90 (70.3%)).

Some patients with very severe strokes were excluded if they could not give informed consent or if no carer or IW could be found to provide assent on their behalf. Consequently, the study cohort had lower mean NIHSS scores than all stroke patients admitted during the data collection periods (*p* < 0.001). Interviewed patients were also slightly less likely to have been admitted on weekends (*p* = 0.04) due to weekday-only researcher availability and a high patient turnover on the unit. However, mean age and sex distribution were similar between the interviewed cohort and the larger population admitted during the same period.

### 3.2. Help-Seeking Response

**Figure 1:**
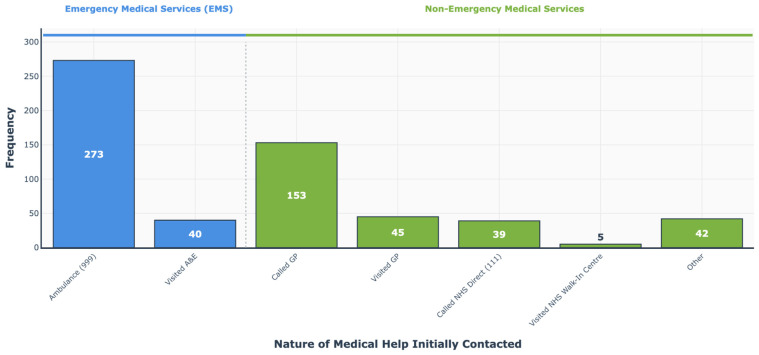
Initial help-seeking response following symptom onset.

In total, 312 (52.4%) patients dialled 999 or attended the Emergency Department as their first contact with medical services following symptom onset. The remainder contacted their GP (31.1%), dialled NHS 111 (6.6%), or contacted another agency (7.1%) (Figure 1).

In 455 (75.6%) cases, an IW called for help on the patient’s behalf. Sometimes, this was done in collaboration with the patient. However, in 282 (46.8%) cases, the IWs were “solely responsible” for calling for help, e.g., in cases where the patient was incapacitated by their symptoms or had refused to seek medical help.

Overall, the median (IQR) time to first call for any help was 49.8 (10.2–402.9) minutes. The calls were made significantly faster in those who called EMS first (25.2 (4.8–90.0) minutes) versus those who did not (4 h (30–140 min)) (*p* < 0.001).

Patients for whom EMS was called first were more likely to arrive within 4 h of symptom onset (59.3%) than when other agencies were called (20.3%) (*p* < 0.001).

Table 2 outlines sociodemographic, clinical, and knowledge factors associated with the decision to call EMS as opposed to a non-emergency route. These relationships are described in more detail below.

### 3.3. Demographic and Clinical Factors Associated with EMS Use

The mean (SD) age of the patients who contacted EMS in the first instance was 73.8 (14.3) compared with 70.0 (13.9) in those who did not, corresponding to a mean age difference of 3.85 years (95% CI 1.57–6.12; *p* = 0.001). There were no significant associations between dichotomised sex, educational attainment, socioeconomic status, or weekend symptom onset and direct EMS calls (Table 2).

Greater stroke severity (NIHSS ≥ 5) was associated with an increased likelihood of EMS contact (adjusted OR 1.92, 95% CI 1.18–3.14; *p* = 0.009). The presence (versus absence) of FAST symptoms (facial weakness, arm weakness, or speech disturbance) was associated with increased EMS use on univariate analysis, but not after multivariable adjustment (*p* = 0.15) (Table 2).

The presence of dysarthria, balance difficulties, and confusion (Table 2) were each independently associated with higher odds of contacting EMS. In contrast, there were no associations between initial EMS contact and facial weakness, facial numbness, arm weakness, arm numbness, leg weakness, diplopia, dysphasia, monocular visual loss, hemianopia, pain, or headache.

### 3.4. Knowledge Factors Associated with Help-Seeking

Only 145 (24.1%) patients correctly attributed their symptoms to stroke/TIA at onset. However, this was not associated with an increased likelihood of calling EMS directly (adjusted OR 1.43, 95% CI 0.84–2.45) (Table 2).

**Figure 2:**
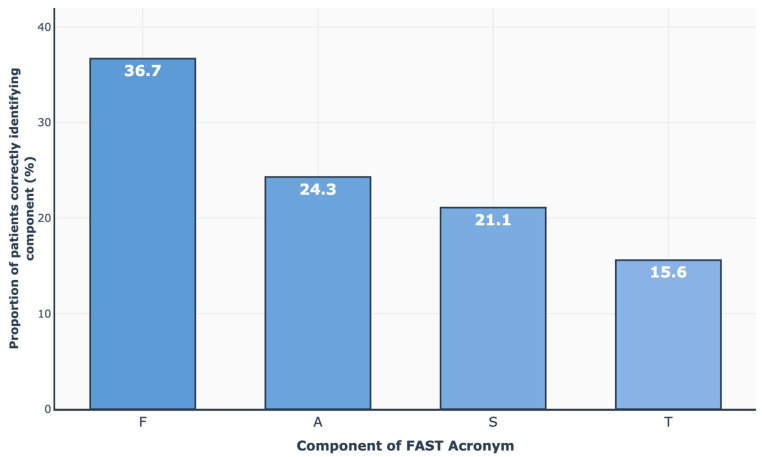
Proportion of patients correctly identifying each component of the FAST acronym (Face, Arm, Speech, Time).

While 469 (77.9%) patients stated that they had been aware of stroke media campaigns prior to their stroke/TIA, only 374 (62.1%) had heard of the acronym “FAST”, and only 208 (34.5%) could accurately name any of the FAST components when prompted. Correct recall decreased sequentially as follows: F (36.7%), A (24.3%), S (21.1%), T (15.6%) (Figure 2). Nevertheless, the ability to recall one or more FAST components was not associated with increased EMS use in this cohort, although this finding should be interpreted with caution given potential selection bias and residual confounding (adjusted OR 0.88, 95% CI 0.52–1.48, *p* = 0.62).

Only 55 (9.1%) patients were aware of any time-critical treatments for stroke.

### 3.5. Barriers to Help-Seeking

The list of potential barriers to help-seeking, derived from previously published studies in stroke/elderly cohorts, resonated with most participants, and were reported by 463 (81.2%) patients and 64 (53.3%) IWs (*p* < 0.001) (Table 3). These barriers were frequently present even when the person calling for help had correctly recognised the symptoms as potentially being due to a stroke (Table 3). For example, “waiting to see if symptoms resolved” and “not thinking symptoms were serious” were reported by 39% and 29.5% of patients who had correctly attributed symptoms to stroke, respectively (Table 3). Both of these barriers were independently associated with reduced likelihood of contacting EMS after adjusting for NIHSS score (adjusted OR 0.34, 95% CI 0.21–0.55, *p* < 0.001, and adjusted OR 0.52, 95% CI 0.32–0.84, *p* = 0.007, respectively) (Table 4).

### 3.6. Thrombolysis Rates

Eighteen (4.2%) stroke patients received intravenous thrombolysis. A further 170 stroke patients were potentially eligible for thrombolysis (having no other apparent contraindications upon records review) had they arrived within the therapeutic time window.

## 4. Discussion

### 4.1. Principal Findings

Despite widespread public awareness of the existence of the stroke media campaign, this study suggests a disconnect between knowledge and action. While 77.9% of patients reported having seen the stroke symptom awareness campaigns, only 24.1% correctly attributed their symptoms to stroke at onset. More importantly, correct symptom recognition was not associated with appropriate help-seeking behaviour, as patients who identified their symptoms as stroke were no more likely to call emergency services directly than those who did not. This finding challenges the fundamental assumption underlying current public health campaigns: that education about stroke symptoms will drive urgent action. These findings support those of Dombrowski et al [20], who led a UK study to examine the effect of an ACT FAST leaflet on public response times. In that study, those who had received the FAST information leaflet recalled more FAST signs (75.7% vs. 68.2%; *p* = 0.003) but showed no improvement in stroke recognition or intended emergency response compared to controls [20].

Our study identified two key barriers that were independently associated with failure to contact emergency medical services: not perceiving symptoms as serious and waiting to see if symptoms would resolve. Notably, these barriers were present even among patients who had initially attributed their symptoms to stroke or TIA, with 29.5% reporting that they did not think symptoms were serious and 38.8% reporting that they waited for symptom resolution (Table 3). This suggests that improving symptom recognition alone addresses only part of the problem, and that perceived severity may play a critical role in help-seeking behaviour. These findings are consistent with the analysis by Teuschl et al. (2010), who similarly reported that the decision to seek emergency care following stroke onset is more strongly influenced by perceived symptom severity rather than by knowledge of their causation [21].

The critical missing element appears to be knowledge and/or understanding of emergency treatments, their potential benefits, and their time-critical nature.

### 4.2. Barriers to Help-Seeking: Beyond Symptom Recognition

Whilst many patients simply did not realise they were having stroke symptoms, among those who correctly attributed their symptoms to stroke, 39% waited to see if symptoms would resolve and 29.5% did not perceive them as serious. We found that both behaviours were independently associated with reduced emergency services utilisation, even after controlling for stroke severity. Similarly, Iversen et al. (2020) found that both patients and bystanders who perceived the situation as “very serious” were significantly more likely to make a primary EMS contact, arrive within 3 h, and receive reperfusion therapy, independent of stroke severity and age [22]. The fact that patients adopted a “wait-and-see” approach, even when they suspected stroke, suggests that even when public education has successfully conveyed what a stroke looks like, it may have failed to communicate why immediate action is critical.

The role of intervening witnesses adds complexity to this picture. It was noted that whilst witnesses were more likely to correctly identify stroke symptoms (55.0% versus 24.6%), they still reported substantial barriers to seeking help, albeit fewer than the patients who made their own call-outs (53.3% versus 81.2%, *p* < 0.001). Previous studies have demonstrated that the presence of a bystander and knowledge of two or more stroke symptoms more than doubled the odds of a prompt EMS call [22]. Taken together, these findings suggest that bystanders may be a valuable target for public education, as they are frequently involved in accessing help and appear more capable of correctly recognising stroke symptoms, yet still require messaging about the urgency of calling EMS.

### 4.3. Clinical Symptom Patterns and Help-Seeking

The presence of dysarthria (slurred speech) was the only FAST symptom independently associated with emergency services contact after adjusting for stroke severity. There are several potential reasons for this. Dysarthria may be alarming to both the patient and the witnesses, and it interferes with communication, often necessitating third-party help. Some other classical posterior circulation symptoms also showed independent associations with emergency services utilisation, e.g., balance impairment, vertigo and confusion. These symptoms may therefore have been perceived as more serious or alarming than isolated weakness or numbness in this cohort. Previous research has called for expanding public awareness of stroke to include posterior circulation symptoms. For example, a UK study of minor strokes noted that nearly one-third presented with visual symptoms (blurred or lost vision), leading the authors to propose adding “Eyes” to the FAST acronym [23]. Furthermore, U.S. hospitals have proposed the “BE FAST” acronym (Balance, Eyes, Face, Arm, Speech, Time) to capture posterior circulation strokes. Early reports indicate that adding balance/vision can reduce missed stroke diagnoses [24].

### 4.4. The Role of Primary Care in Stroke Pathways

In our study, nearly one-third of patients (31.1%) called their GP as the first point of access, and an additional 7.5% visited their GP in person. This represents a substantial cohort entering inappropriate care pathways, which likely contributed to delays in them receiving definitive treatment. Previous research confirms that this pattern is widespread: one study found 29% of stroke patients initially sought GP care, with only 39% subsequently transferred to hospital by emergency services [25].

The primary care bottleneck likely reflects multiple factors: longstanding relationships with GPs leading patients to seek familiar care, telephone triage systems that may not recognise atypical presentations, and lack of immediate appointment availability forcing patients to wait. While GPs can facilitate rapid transfer for obvious strokes, our data suggest many patients with less obvious stroke presentations experience delays. This represents a critical gap in the care pathway that future interventions should address.

### 4.5. Knowledge Gaps: Poor Recall of T for Time

The poor recall of the “time” component of FAST (15.6% correct recall, compared to 36.7% for “face”) is particularly concerning given that availability of time-critical treatments in hyperacute stroke centres is the rationale for emergency action. Only 9.1% of patients in our cohort were aware of any time-dependent treatments, and just 6.6% could name thrombolysis specifically or refer to a “clot-busting” treatment.

In some international studies, a higher awareness of thrombolysis (20.8% in Minnesota, 18% in Republic of Korea) has been reported [26,27]. One response to this discrepancy could be to reinforce the “time” component of FAST in the UK campaigns. However, whilst this prompts people to call 999 immediately, the campaign message omits to mention the rationale for the urgency. Messages emphasising reperfusion time dependency, such as “treatments within hours prevent disability”, could provide clearer motivation for immediate action. Visual content depicting clot removal or documented recovery trajectories could make the time-critical nature of stroke more compelling and memorable.

### 4.6. Comparison with Existing Literature

Our findings align with and extend previous research. Multiple studies have documented the gap between stroke knowledge and appropriate behaviour [20,25,28], but most have focused on demographic factors or stroke characteristics as factors associated with emergency services use [25,28,29]. Smaller qualitative studies have explored cognitive and emotional barriers [10,11,18,19,30], but lacked statistical power to quantify their relative importance. Our study bridges this gap by measuring barrier prevalence in a large sample and establishing their independent associations with the desired help-seeking behaviour (i.e., a rapid call to EMS).

One previous study (*n* = 149) examined barriers to help-seeking in relation to the time delay between symptom onset and arrival at hospital [31]. However, hospital arrival time confounds patient decision-making with system factors (ambulance response times, emergency department processing), making it less useful as an outcome measure of behavioural interventions. We argue that emergency services (EMS) utilisation as the initial first contact is a superior outcome measure because it directly reflects patient decision-making and can be objectively verified through ambulance records.

### 4.7. Clinical and Policy Implications

Our findings have several implications for practice and policy. First, future public education campaigns must move beyond symptom recognition to emphasise three key messages: (1) stroke symptoms require immediate emergency services contact, (2) waiting to see if symptoms improve wastes critical treatment time, and (3) highly effective treatments exist but are only available if patients reach hospital quickly. These messages should have targeted channels through television and social media.

Second, we suggest that the “FAST” acronym requires enhancement or supplementation. While it successfully promotes symptom recognition in some cases, it needs to convey treatment availability and its time-critical nature. A revised campaign might use concrete examples: “treatments can remove the blood clot and restore brain function, but only if given within hours” or “every minute counts brain cells die quickly without blood flow”.

Third, there may be scope to enhance the role of primary care in stroke pathways. For example, when patients contact their GP surgery with possible stroke symptoms, the practice needs clear protocols to direct them straight to emergency services, rather than telephone triage or elective booking of an appointment. If a patient describes sudden neurological symptoms, the call should go directly to 999, skipping the usual assessment steps entirely. At the same time, public campaigns should make it clear that stroke symptoms warrant calling 999 directly, not the GP surgery. It is then for the call handler to determine the appropriate level of response and guide the patient/IW accordingly.

Finally, we found that several non-FAST symptoms, including balance impairment and confusion, were independently associated with emergency medical services contact (Table 2). This suggests that patients and witnesses already recognise these symptoms as serious warning signs. This highlights an opportunity to reinforce the urgency of these symptoms in public health campaigns, either by integrating them with FAST criteria or presenting them as additional warning signs that warrant immediate action.

It is worth noting that data were collected between 2013 and 2016. While the behavioural processes underlying help-seeking are unlikely to have fundamentally changed, advances in stroke care, including expanded thrombectomy time windows and evolving service delivery, may influence the contemporary applicability of these findings.

### 4.8. Strengths and Limitations

Our study’s strengths include its large sample size, systematic assessment of barriers using validated instruments, inclusion of both patients and intervening witnesses, and use of objective outcome measures verified against ambulance records. The 72 h interview window minimised recall bias while ensuring patients had recovered sufficiently to participate.

Several limitations require consideration. First, retrospective assessment of barriers introduces potential recall bias, although the short interview window mitigates this concern. Second, we did not interview bystanders who had witnessed symptoms but did not call for help, therefore limiting generalisability of findings about witnesses or bystanders in general.

Third, we found no association between FAST awareness and help-seeking behaviour, but cannot conclude FAST has been ineffective, as we lack counterfactual data about what the same patients would have done without any prior exposure to stroke campaigns. Fourth, convenience sampling during weekday working hours meant our sample was not consecutive. This resulted in underrepresentation of weekend admissions. In addition, some patients with severe strokes had to be excluded if too unwell to consent. In view of the rapid turnaround of TIA patients, there were also some TIA patients who went home straight after clinic before they could be interviewed (e.g., if the researcher was interviewing another participant). As a result, the study population was skewed towards patients with milder stroke severity, which may limit the applicability of findings to patients with more severe stroke presentations, where barriers to help-seeking may differ. However, there was no systematic bias in inclusion of patients admitted during researcher hours, and the large size of our sample allowed for any potential biases to be accounted for in the subgroup analyses.

Fifth, community TIA patients who never presented to medical services are not represented in our sample. That said, there were 79 patients who admitted to having prior TIA-like episodes in the preceding three months, nearly half of whom (45.6%) did not seek help at the time of the first event, suggesting we captured at least some of this population. Sixth, we combined emergency services calls with emergency department walk-ins in our definition of “EMS use”. While ambulance transport is preferable for speedy conveyance, both behaviours demonstrated a recognition of the “emergency” nature of the symptoms, which is why we pooled the two categories. This may introduce heterogeneity, as ambulance use more directly reflects emergency response behaviour. Seventh, we did not collect detailed vascular risk factor data, prior stroke or cardiovascular disease history, or family history, which may influence help-seeking behaviour. Eighth, thrombolysis eligibility estimates were based on retrospective notes review and did not include systematic assessment of all potential contraindications, and may therefore overestimate true eligibility. Finally, we did not assess the timing, frequency, or recency of individual exposure to stroke awareness campaigns, and therefore cannot account for potential variation in campaign exposure at the time of symptom onset. In addition, for wake-up strokes, time of first symptom awareness was used as a proxy for onset time, which may underestimate true delay and influence analyses of help-seeking behaviour. Finally, the study was conducted within a single hyperacute stroke centre in the North of England, which may limit generalisability to other settings.

## 5. Conclusions

Our study highlights a potential public health gap between levels of knowledge of stroke, correct recognition of symptoms, and decisions to contact emergency services when stroke symptoms occur. There was a tendency for patients with stroke symptoms, and for other people witnessing them, to “wait and see” if the symptoms would resolve and to deprioritise them. Recall of the “Time” component of the FAST campaign message was also relatively poor. Incorporating the rationale for seeking fast access to hospital (i.e., that time-critical treatments can avoid a lifetime of disability) could encourage more people to utilise emergency services and has the potential to significantly increase access to thrombolysis and thrombectomy.

## Figures and Tables

**Table 1:** Baseline characteristics of patients and intervening witnesses.

Characteristic	Patients (%)	Intervening Witnesses (%)
**Sociodemographic Factors**		
Age, mean (SD), Years	71.8 (14.2)	64.7 (16.9)
Sex		
Male sex	325 (54.2)	38 (30.9)
Ethnicity		
White	569 (96.1)	113 (92.6)
Pakistani	12 (2.0)	5 (4.1)
Black	10 (1.7)	3 (2.5)
Other	1 (0.2)	1 (0.8)
Basic education only	371 (67.8)	66 (58.4)
Most deprived 2 quintiles (IMD)	274 (46.3)	38 (44.2)
**Clinical Factors**		
Diagnosis		
Stroke	429 (71.3)	—
TIA	173 (28.7)	—
Time of event		
Weekday	451 (74.9)	91 (71.1)
Weekend	151 (25.1)	37 (28.9)
FAST symptoms at onset	457 (77.2)	—
Number of FAST symptoms at onset		
0	160 (29.7)	—
1	181 (33.6)	—
2	115 (21.3)	—
3	83 (15.4)	—
Medical help initially contacted		
EMS (999 call)	272 (45.2)	70 (54.7)
Called GP	152 (25.2)	38 (29.7)
Visited GP	45 (7.5)	1 (0.8)
NHS Direct Helpline (111)	39 (6.5)	7 (5.5)
Emergency department attendance	40 (6.6)	4 (3.1)
Walk-in centre	5 (0.8)	0 (0)
Other	42 (7.0)	4 (3.1)
Arrival in <4 h of symptom onset	184 (32.6)	—
NIHSS, median (IQR)	3 (1–6)	—
Thrombolysed *	18 (4.2)	—
**Knowledge Factors**		
Correct perception of symptoms	145 (24.6)	66 (55.0)
Able to name ≥1 component of FAST	210 (37.3)	70 (58.3)
FAST components recalled		
0	353 (62.7)	50 (41.7)
1	52 (9.2)	10 (8.3)
2	50 (8.9)	12 (10.0)
3	37 (6.6)	20 (16.7)
4	71 (12.6)	28 (23.3)
Aware of time-based treatments	55 (9.1)	16 (12.5)

* Of stroke patients only. IMD = Index of Multiple Deprivation; NIHSS = National Institutes of Health Stroke Scale; TIA = transient ischaemic attack; FAST = Face, Arm, Speech, Time; EMS = emergency medical services; GP = general practitioner; NHS = National Health Service.

**Table 2:** Factors associated with emergency medical services (EMS) contact.

	EMS Contacted (Figures Represent *n* (%) Unless Otherwise Stated)		Odds Ratio (95% CI)	*p*-Value	Adjusted Odds Ratio * (95% CI)	*p*-Value
	Yes	No				
**Sociodemographic Factors**						
Mean age (SD), years	73.8 (14.3)	70.0 (13.9)	—	**0.001**	—	—
Age ≥ 75 years	155 (57.2)	131 (40.8)	1.94 (1.40–2.70)	**<0.001**	2.35 (1.61–3.42)	**<0.001**
Male sex	138 (50.9)	184 (57.1)	0.78 (0.56–1.08)	0.13	0.87 (0.60–1.26)	0.47
Basic education only	179 (72.8)	191 (63.9)	1.51 (1.05–2.18)	**0.03**	1.35 (0.88–2.07)	0.17
Most deprived two quintiles (IMD)	125 (46.8)	146 (45.9)	1.04 (0.75–1.44)	0.83	1.03 (0.71–1.49)	0.87
**Clinical Factors**						
Facial weakness at onset	135 (50.6)	83 (26.3)	2.87 (2.03–4.06)	**<0.001**	1.47 (0.86–2.49)	0.16
Facial numbness at onset	57 (21.3)	49 (15.4)	1.49 (0.98–2.27)	0.06	1.22 (0.73–2.01)	0.45
Arm weakness at onset	143 (53.4)	117 (36.7)	1.98 (1.42–2.75)	**<0.001**	1.20 (0.79–1.83)	0.39
Arm numbness at onset	70 (26.5)	76 (23.9)	1.15 (0.79–1.67)	0.47	0.97 (0.63–1.51)	0.90
Hand weakness at onset	70 (26.6)	76 (23.9)	1.16 (0.79–1.68)	0.45	1.03 (0.68–1.55)	0.90
Hand numbness at onset	126 (47.4)	112 (35.1)	1.66 (1.19–2.32)	**0.003**	1.01 (0.65–1.58)	0.96
Leg weakness at onset	127 (47.2)	117 (36.7)	1.54 (1.11–2.15)	**0.01**	1.03 (0.69–1.53)	0.89
Leg numbness at onset	65 (24.5)	61 (19.2)	1.37 (0.92–2.03)	0.12	1.26 (0.80–1.98)	0.33
Dysphasia at onset	99 (36.8)	81 (25.4)	1.71 (1.20–2.44)	**0.003**	1.26 (0.84–1.87)	0.26
Dysarthria at onset	161 (59.6)	114 (35.7)	2.66 (1.90–3.71)	**<0.001**	1.89 (1.26–2.82)	**0.002**
Balance impairment at onset	116 (43.3)	98 (30.7)	1.72 (1.23–2.42)	**0.002**	1.97 (1.34–2.90)	**<0.001**
Vertigo at onset	68 (25.4)	54 (16.9)	1.67 (1.12–2.49)	**0.01**	2.00 (1.27–3.15)	**0.003**
Diplopia at onset	24 (9.0)	50 (15.7)	0.53 (0.31–0.89)	**0.01**	0.77 (0.43–1.37)	0.37
Monocular visual loss at onset	14 (5.2)	29 (9.1)	0.55 (0.28–1.06)	0.07	0.54 (0.25–1.19)	0.13
Hemianopia at onset	18 (6.7)	25 (7.9)	0.84 (0.45–1.58)	0.59	0.81 (0.37–1.75)	0.59
Headache at onset	85 (31.7)	93 (29.2)	1.13 (0.79–1.61)	0.50	1.38 (0.91–2.09)	0.13
Confusion at onset	55 (39.3)	37 (23.0)	2.17 (1.32–3.58)	**0.002**	2.05 (1.13–3.73)	**0.02**
Other pain at onset	16 (6.0)	28 (8.8)	0.66 (0.35–1.25)	0.20	0.90 (0.44–1.84)	0.90
Diagnosis stroke (versus TIA)	200 (73.5)	223 (69.0)	1.25 (0.87–1.78)	0.23	0.75 (0.49–1.14)	0.18
Any ‘FAST’ symptoms at onset	232 (85.9)	223 (69.9)	2.63 (1.73–3.99)	**<0.001**	1.08 (0.62–1.88)	0.79
Episode occurred over a weekend	74 (27.2)	72 (22.3)	1.30 (0.90–1.89)	0.17	1.22 (0.79–1.87)	0.37
**Knowledge Factors**						
Had attributed symptoms to stroke/TIA	74 (27.5)	70 (22.0)	1.34 (0.92–1.96)	0.12	1.72 (1.11–2.65)	**0.02**
Could name ≥ 1 FAST component	86 (34.7)	124 (39.9)	0.80 (0.57–1.13)	0.21	0.91 (0.61–1.37)	0.66
Aware of time-based treatments	31 (11.9)	46 (14.6)	0.79 (0.49–1.29)	0.35	0.69 (0.40–1.20)	0.19

* Adjusted for age, sex, and number of FAST symptoms. Bold values indicate *p* < 0.05. Female sex used as the reference category.

**Table 3:** Barriers to help-seeking reported by patients and intervening witnesses.

	Patients				Intervening Witnesses			
Initial Perceived Cause of Symptoms					Initial Perceived Cause of Symptoms			
Barrier to Help-Seeking	Stroke or TIA *n* (%)	Other (%)	Total (%)	*p*-Value	Stroke or TIA *n* (%)	Other *n* (%)	Total (%)	*p*-Value
Did not think symptoms were serious	41 (29.5)	233 (55.0)	274 (48.7)	**<0.001**	6 (9.5)	17 (32.1)	23 (19.8)	**0.002**
Believed in fate	32 (23.0)	80 (18.9)	112 (19.9)	0.29	1 (1.6)	4 (7.5)	5 (4.3)	0.12
Worried about troubling others	50 (36.0)	184 (43.2)	234 (41.4)	0.13	3 (4.8)	2 (3.8)	5 (4.3)	0.79
Waited to see if symptoms would resolve	54 (38.8)	230 (54.0)	284 (50.3)	**0.002**	5 (7.9)	12 (22.6)	17 (14.7)	**0.03**
Embarrassed to call 999	26 (19.0)	91 (21.4)	117 (20.8)	0.54	2 (3.2)	1 (1.9)	3 (2.6)	0.66
Fear of hospitals/tests	15 (10.8)	34 (8.0)	49 (8.7)	0.31	1 (1.6)	4 (7.5)	5 (4.3)	0.12
Symptoms were low priority	16 (11.5)	62 (14.6)	78 (13.8)	0.37	1 (1.7)	3 (5.8)	4 (3.6)	0.26
Did not think anything could be done to treat	16 (11.7)	21 (4.9)	37 (6.6)	**0.005**	3 (4.8)	2 (3.8)	5 (4.3)	0.79

Values are *n* (%). Bold *p*-values indicate *p* < 0.05. Patients *n* = 570, intervening witnesses *n* = 120.

**Table 4:** Relationship between barriers to help-seeking and emergency medical services (EMS) contact.

	EMS Contacted?		Odds Ratio (95% CI)	*p*-Value	Adjusted Odds Ratio * (95% CI)	*p*-Value
Barrier to Help-Seeking	Yes *n* (%)	No *n* (%)				
Did not think symptoms were serious	94 (37.3)	180 (57.5)	0.44 (0.31–0.62)	**<0.001**	0.42 (0.29–0.62)	**<0.001**
Believed in fate	56 (22.2)	56 (17.9)	1.31 (0.87–1.99)	0.20	1.54 (0.95–2.48)	0.08
Worried about troubling others	92 (36.4)	141 (44.9)	0.70 (0.50–0.98)	**0.04**	0.70 (0.48–1.03)	0.07
Waited to see if symptoms would resolve	91 (36.0)	192 (61.1)	0.36 (0.25–0.50)	**<0.001**	0.37 (0.25–0.54)	**<0.001**
Embarrassed to call 999	51 (20.2)	66 (21.2)	0.94 (0.62–1.41)	0.76	1.10 (0.69–1.76)	0.69
Fear of hospitals/tests	28 (11.1)	21 (6.7)	1.74 (0.96–3.14)	0.07	1.79 (0.91–3.52)	0.09
Symptoms were low priority	24 (9.5)	55 (17.5)	0.49 (0.30–0.82)	**0.01**	0.43 (0.24–0.76)	**0.003**
Did not think anything could be done to treat	15 (6.0)	22 (7.0)	0.84 (0.43–1.67)	0.62	0.83 (0.37–1.87)	0.66

* Adjusted for age, sex, and number of FAST symptoms. Bold *p*-values indicate *p* < 0.05.

## Data Availability

The raw data supporting the conclusions of this article will be made available by the authors on request.

## Supplementary Materials

The following supporting information can be downloaded at: https://www.mdpi.com/article/10.3390/neurosci7030070/s1.
