# Supplementary material for: Barriers Associated with Help-Seeking for Stroke Symptoms Despite Public Awareness Campaigns: A Cross-Sectional Study
Source: NeuroSci. 2026 Jun 14;7(3):70. doi: 10.3390/neurosci7030070 (PMC13305497; doi:10.3390/neurosci7030070)
Supplement: Supplementary file 1 [file neurosci-07-00070-s001.zip › neurosci-4275198-supplementary.pdf]

Stroke and TIA Awareness and Response to Symptoms study (STARS)

Study ID no: STH17133

Name:
DoB: (Affix Patient Label Here)
Hosp No.:
NHS No.:
Consultant:

Patient ID number ☐ ☐ ☐

Name ____

DoB __ / __ / ________

Hospital number _________

Gender: ☐ Male ☐ Female

Address ____

____

____

Telephone 1 _____________________

Telephone 2 _____________________

Date of admission ____/____/________

Date of interview ____/____/________

Patient ID number ☐ ☐ ☐

Sheffield Teaching Hospitals NHS
NHS Foundation Trust

---

1. Did an interview with the patient take place? ☐ Yes ☐ No ☐ Unknown

---

2. If no, why not? (tick all that apply)

☐ Refused ☐ Aphasic ☐ Unwell ☐ Dementia

☐ Unable to contact patient ☐ Other ____

---

3. How was the interview conducted?

☐ Face-to-face ☐ Telephone

---

4. Who was the information obtained from?

☐ Patient ☐ Relative (state relationship) ____________________________

☐ Spouse ☐ Paid Carer ☐ Hospital Records

☐ Other ________________

---

5. What is the current diagnosis?

☐ Definite Stroke ☐ Probable Stroke

☐ Definite TIA ☐ Probable TIA

---

6. If your symptoms have resolved, how long did your symptoms last in total?

Days_______ Hours ______ Minutes _______ ☐ N/A (symptoms continue)

---

7. Did the stroke/TIA occur whilst you were in hospital for another reason? ☐ Yes ☐ No

8. Were you transferred from another hospital? ☐ Yes ☐ No

Please state which hospital ____

---

9. Patients first ever stroke/TIA? ☐ Yes ☐ No

Which symptoms did you have at the ONSET of your stroke/TIA?

Weakness

10. Face	☐ Yes	☐ No	☐ Right	☐ Left
11. Arm	☐ Yes	☐ No	☐ Right	☐ Left
12. Leg	☐ Yes	☐ No	☐ Right	☐ Left
13. Hand	☐ Yes	☐ No	☐ Right	☐ Left

Loss of sensation

14. Face	☐ Yes	☐ No	☐ Right	☐ Left
15. Arm	☐ Yes	☐ No	☐ Right	☐ Left
16. Leg	☐ Yes	☐ No	☐ Right	☐ Left
17. Hand	☐ Yes	☐ No	☐ Right	☐ Left

Change of vision

18. Loss of outer half field of vision in one eye and inner half of field of vision in other eye (hemianopia)	☐ Yes	☐ No
19. Loss of vision confined to one eye (monocular)	☐ Yes	☐ No
20. Double vision (diplopia)	☐ Yes	☐ No

Change in speech

21. Word finding difficulties (dysphasia)	☐ Yes	☐ No
22. Slurred speech (dysarthria)	☐ Yes	☐ No

Other symptoms

23. Spinning sensation (vertigo)	☐ Yes	☐ No
24. Headache	☐ Yes	☐ No
25. Pain	☐ Yes	☐ No
26. Imbalance	☐ Yes	☐ No

Presenting event

27. Who first noticed you had the symptoms?

☐ You ☐ Spouse ☐ Relative ☐ Friend

☐ Neighbour ☐ Stranger Other ______________________________________

---

28. What time did you (or someone else) first notice you had symptoms?

Date ____/____/________ Time ____:____

---

29. Where were you when you (or someone else) first noticed you had symptoms?

☐ Home ☐ Work ☐ Leisure(non-sport)

☐ Sport Other __________________________________

---

30. Did you first notice your symptoms on waking from sleep? ☐ Yes ☐ No (go to question 33)

---

31. If so, what time did you wake up with the symptoms? Time ____:____

---

32. If so, what was the date/time that you were last well (free of the stroke/TIA symptoms)?

Date ____/____/________ Time ____:____

---

33. What were you doing at the time that you (or someone else) first noticed that you had symptoms?

………

………

---

34. Were you alone when your symptoms started? ☐ Yes ☐ No

---

35. If you were with someone when your symptoms started, who was it?

☐ Spouse ☐ Friend ☐ Relative

☐ Colleague Other______________________________________

---

36. What did you **first** think was wrong?

☐ Stroke ☐ TIA ☐ Migraine ☐ Heart Attack

☐ Didn't Know ☐ Can't Remember Other______________________________

---

37. Did you call for help (medical or non-medical) immediately? ☐ Yes ☐ No

---

38. If you did **not** call for help (medical or non-medical) immediately, why not?
(Interviewer to prompt - any other reasons?)

……

……

……

……

---

39. If you **did** call for help (medical or non-medical) immediately, why did you?
(Interviewer to prompt - any other reasons?)

……

……

……

……

---

40. Who was the first person you either told about your symptoms, or who first became aware of your symptoms?

☐ Spouse ☐ Friend ☐ Relative ☐ GP

☐ 999 ☐ A&E (you drove to hospital yourself) ☐ NHS walk-in

☐ Neighbour Other______________________________

---

41. What was the reaction/s of the first person (medical or non-medical) who became aware of your symptoms?

☐ They encouraged me to call 999 ☐ They called 999 for me

☐ They encouraged me to call GP ☐ They called the GP for me

☐ They encouraged me to go to A&E ☐ They came to see me

☐ They drove me to A&E

☐ They drove me to a local clinic

☐ They recommended I wait to see if my symptoms improved

Other ____

---

42. Who first realised your symptoms could be due to an illness?

☐ You ☐ Spouse ☐ Friend ☐ Relative

☐ GP ☐ 999 operator ☐ Staff at NHS walk-in

☐ Neighbour Other______________________________

---

43. When did you (or someone else) first realise your symptoms could be due to an illness?

Date ____/____/________ Time ____:____

---

44. When did you (or someone else if you were unable) first ask for the help/advice of another person/agency? (medical or non-medical)

Date ____/____/________ Time ____:____

---

45. Who was the person responsible for calling **medical** help?

☐ You ☐ Spouse ☐ Friend ☐ Relative (state relationship) __________________

☐ Neighbour ☐ Other_____________________________

---

46. If someone else was responsible for calling for medical help, were they the **only** person responsible for the call for medical help? (see question 45 for example scenarios)

☐ Yes *(recruit that person to the study and ask them to complete QA V.2)*

☐ No (go to question 48)

---

47. Why was that other person solely responsible for calling for medical help?

☐ Patient refused to call for help

☐ Patient asked you to make the decision on their behalf

☐ Patient incapacitated by stroke/TIA and unable to make call

☐ Patient incapacitated for another reason and unable to make call

Other ____

---

48. Which **medical** help was the first contacted?

☐ 999 ☐ GP by telephone ☐ GP in person ☐ NHS direct telephone

☐ A&E in person ☐ NHS walk-in Other____________________________________

---

49. If this was not 999/A&E in person, why not?

………

………

---

50. When was the first call for medical help made?

Date ____/____/________ Time ____:____

---

51. If ambulance was called, what date and time?

Date ____/____/________ Time ____:____

---

52. What was the outcome of the first call for medical assistance?

☐ I made an appointment with GP

☐ I was advised to call an ambulance

☐ A GP came to the house

☐ I was told to go to A&E

☐ Ambulance came

Other ____

---

53. Which type of medic did you see first? ☐ Hospital doctor ☐ GP

☐ Paramedic ☐ Nurse

---

54. When did you first see a medic?

Date ____/____/________ Time ____:____

---

55. How were you transported to the emergency department/hospital?

☐ Ambulance ☐ Personal car ☐ Relatives/friends car

☐ Taxi ☐ Public Transport Other_____________________________________

---

56. When did you arrive at the stroke admissions unit?

Date ____/____/________ Time ____:____

Ambulance call-outs (see yellow ambulance sheet)

57. When was the ambulance call made (originated)?

Date ____/____/________ Time ____:____ ☐ N/A

58. When did the ambulance arrive at the scene?

Date ____/____/________ Time ____:____ ☐ N/A

59. When did the ambulance leave the scene?

Date ____/____/________ Time ____:____ ☐ N/A

60. When did the ambulance arrive at the hospital?

Date ____/____/________ Time ____:____ ☐ N/A

Recent stroke/TIAs

61. Have you had any episodes of stroke symptoms between the first episode (which resulted in you seeking a medical opinion) and entry into this study? ☐ Yes ☐ No (go to question 64)

62. If yes, how many episodes did you have between the first episode and seeing the first medical person? ______

63. If yes, how many events did you have between seeing the first medical person and entry into this study? ______

64. Have you had any episodes of stroke symptoms in the last 3 months? ☐ Yes ☐ No

65. If so, how many? ______

66. Did you seek medical help for those episodes? ☐ Yes ☐ No

67. If not, why not? …………………………………………………………………………………………

……..

Previous stroke / TIA

68. Have you had a previous stroke? ☐ Yes ☐ No (go to question 72)

69. If yes, how many? ______

70. When was your first stroke? Date ____/____/________

71. When was your most recent stroke? Date ____/____/________

72. Have you had a previous TIA? ☐ Yes ☐ No (go to question 78)

73. If yes, how many? ______

74. When was your first TIA? Date ____/____/________

75. When was your most recent TIA? Date ____/____/________

76. How long did your most recent TIA last? ______ hours ______ minutes

77. How long did your longest TIA last? ______ hours ______ minutes

Other past medical history

78. Have you ever had angina? ☐ Yes ☐ No

79. Have you ever had myocardial infarction? ☐ Yes ☐ No

80. Have you ever had sudden blockage in artery in arm or leg, or a burst artery (e.g. aneurysm)? ☐ Yes ☐ No Specify ________________________

81. Have you ever had an irregular heart beat (atrial fibrillation)? ☐ Yes ☐ No

82. Do you have diabetes? ☐ Yes ☐ No

83. Have you ever had high cholesterol? ☐ Yes ☐ No

84. Have you ever had high blood pressure? ☐ Yes ☐ No (go to question 87)

85. What year was your high blood pressure diagnosed? Year ______

86. Are you on treatment for high blood pressure? ☐ Yes ☐ No

87. How often do you go to your GP's practice for blood pressure check?

☐ Less than once per year ☐ Once a year ☐ More than once a year

88. How often do you check your **own** blood pressure at home?

☐ Never ☐ Daily ☐ Weekly ☐ Monthly ☐ Yearly

Medication

89. Which medication were you taking prior to this admission to hospital?

Warfarin	☐ Yes	☐ No
Aspirin	☐ Yes	☐ No
Clopidogrel	☐ Yes	☐ No
Dipyridamole	☐ Yes	☐ No
Statin	☐ Yes	☐ No
Antihypertensive agent	☐ Yes	☐ No

Smoking

90. Do you smoke? ☐ Currently ☐ Ex-smoker ☐ Never ☐ Unknown

91. How many cigarettes do you / did you smoke per day? ______ cigarettes per day

92. How old were you when you started smoking? ______ years old

93. How old were you when you quit smoking? ______ years old ☐ N/A (current smoker)

94. How much alcohol do you drink per week? (one pint beer / glass wine / shot =2 units)

□ None □ 1-14 units □ 14-21 units □ >21 units

Premorbid Rankin score

Please note that question 89-94 relate to the patients abilities **before** this stroke/TIA

95. Did you have any symptoms of any kind (these can include pain, stiffness, weakness, fatigue)?	☐ Yes	☐ No
96. Were you able to look after yourself and carry out normal activities?	☐ Yes	☐ No
97. Did anyone else help pay bills, do shopping, etc.?	☐ Yes	☐ No
98. Did you need someone to help you walk?	☐ Yes	☐ No
99. Did you need help to wash yourself?	☐ Yes	☐ No
100.Did you need help to be lifted out of the bed?	☐ Yes	☐ No

Total score (/6) ______

ABCD2 score (TIA only)

101. Aged 60 or older?	☐ Yes (1)	☐ No (0)
102. BP greater than 140/90 (use first recorded BP post event e.g. from paramedic chart)	☐ Yes (1)	☐ No (0)
103. Weakness	☐ Yes (2)	☐ No (0)
104. Speech disturbance (dysphasia or dysthria) **without** weakness	☐ Yes (1)	☐ No (0)
105. Duration of symptoms ≥60 minutes	☐ Yes (2)	☐ No (0)
106. Duration 10 - 59 minutes	☐ Yes (1)	☐ No (0)
107. Diabetes	☐ Yes (1)	☐ No (0)

108. Total score _____/7

Functional and socioeconomic profile

109. What is your place of residence?

☐ Own home ☐ Home of relative ☐ Home of friend

☐ Social housing ☐ Rented accommodation

☐ Warden housing ☐ Care home Other___________________________________

---

110. Do you live alone? ☐ Yes ☐ No

---

111. If not, who do you live with?

☐ Partner/Spouse ☐ Adult family ☐ Children under 18

☐ Friends Other______________________________________

---

112. Does anyone assist you at home e.g. to clean, wash, dress?

☐ Spouse ☐ Relative ☐ Private carer

☐ Community services ☐ No assistance needed ☐ Unknown

---

113. What is your marital status?

☐ Married ☐ Widow ☐ Single

☐ Separated ☐ Partner ☐ Unknown

---

114. What is your employment status?

☐ Full time ☐ Part time ☐ Caring for home

☐ Unemployed ☐ Unable to work ☐ Retired

☐ Student ☐ Unknown

---

115. What was your most recent occupation (or your spouse's occupation if you are unemployed?)

……………………………………………………………………………………………………

……………………………………………………………………………………………………

---

116. Socioeconomic status (fill in from response above)

☐ Professional ☐ Managerial/technical ☐ Skilled non-manual

☐ Skilled manual ☐ Partly skilled ☐ Unskilled

☐ Armed forces ☐ Unknown

---

117. What is your ethnic origin?

☐ White ☐ Black Caribbean ☐ Black African

☐ Pakistani ☐ Bangladeshi ☐ Chinese

☐ Unknown Other ___________________________________

---

118. How old were you when you left school? ______ years old

---

119. What educational level have you reached?

☐ Basic (GCSE level) ☐ Further (e.g. apprenticeship/NVQ)

☐ Higher (degree) ☐ Pre-GCSE (no exams) ☐ Unknown

Patient knowledge about stroke symptoms prior to this admission

120. Prior to this admission, what did you think the symptoms of a stroke/TIA were?

Tick box only if patient volunteers the symptom – **do not prompt**)

☐ Confusion ☐ Difficulty speaking ☐ Numbness of face/arm/leg

☐ Weakness of face/arm/leg ☐ Abdominal pain ☐ Tingling

☐ Chest pain ☐ Dizziness ☐ Loss of balance

☐ Problems walking ☐ Loss of vision ☐ Breathlessness

☐ Headache ☐ Room spinning (vertigo)

Other ____

---

121. Prior to admission, **which of the following** did you think were symptoms of stroke/TIA? (interviewer to read out full list)

Confusion	☐ Yes	☐ No
Difficulty speaking	☐ Yes	☐ No
Numbness of face/arm/leg	☐ Yes	☐ No
Weakness of face/arm/leg	☐ Yes	☐ No
Abdominal pain	☐ Yes	☐ No
Tingling of both hands	☐ Yes	☐ No
Chest pain	☐ Yes	☐ No
Dizziness	☐ Yes	☐ No
Loss of balance	☐ Yes	☐ No
Problems walking	☐ Yes	☐ No
Loss of vision	☐ Yes	☐ No
Breathlessness	☐ Yes	☐ No
Headache	☐ Yes	☐ No
Room spinning (vertigo)	☐ Yes	☐ No

Patient knowledge about stroke risk factors prior to this admission

122. Prior to this admission, which **medical conditions** did you think increased a persons risk of stroke?

Tick box only if patient volunteers the symptom – **do not prompt**)

☐ High blood pressure	☐ High cholesterol	☐ Diabetes
☐ Previous stroke/TIA	☐ Irregular heart beat (AF)	☐ Previous heart attack

Other ____

Patient knowledge about lifestyle factors prior to this admission

123. Prior to this admission, which **personal habits** did you think increased a person's risk of stroke?

Tick box only if patient volunteers the symptom – **do not prompt**

☐ Smoking ☐ Alcohol ☐ Obesity

☐ Lack of exercise ☐ Stress ☐ High fat diet

Other ____

124. Prior to this hospital admission, where had you heard about the symptoms of a stroke?

☐ Had a stroke/TIA before ☐ Relative/friend had a stroke ☐ TV

☐ Billboards ☐ Radio ☐ Work/Organisational presentation

☐ Magazine ☐ Newspaper ☐ Friend or relative

☐ Doctor or nurse ☐ Lecturer, state where ______________________

☐ Unable to recall

125. Prior to this hospital admission, which, if any, medications did you think were given to patients following a stroke/TIA? (tick those that are volunteered by patient)

☐ Aspirin ☐ Clopidogrel ☐ Warfarin

☐ Statin ☐ Blood pressure lowering drug

Other ____

126. Prior to this admission, which, if any, treatments did you think needed to be given **very quickly in hospital** after a stroke?

☐ Thrombolysis (clot buster) Other ______________________________

127. Did you take any tablets yourself prior to seeking medical help? ☐ Yes ☐ No

128. Where did you hear about these emergency treatments?

☐ TV Advert ☐ Billboards ☐ Radio

☐ Television ☐ Work/Organisational presentation ☐ Magazine

☐ Newspaper ☐ Friend or relative ☐ Doctor or nurse

☐ Unable to recall

129. Prior to this admission were you aware of any media campaign about stroke? ☐ Yes ☐ No
(interviewer can use prompt: e.g. burning face image)

---

130. If yes, where did you see it?

☐ TV Advert ☐ Billboards ☐ Radio

☐ Television ☐ Work/Organisational presentation ☐ Magazine

☐ Newspaper ☐ Notice board, state where ________________________________

---

131. Have you heard of FAST? ☐ Yes ☐ No

---

132. What does it stand for?

……………………………………………………………………………………………………

……………………………………………………………………………………………………

---

133. How did the advert influence you in call for medical help after your symptoms?

☐ Made me call 999 more quickly

☐ Made me call 999 less quickly

☐ Did not influence me in calling for help

Other ____

---

134. Why did the FAST advert affect you in this way?

……………………………………………………………………………………………………

……………………………………………………………………………………………………

……………………………………………………………………………………………………

……………………………………………………………………………………………………

Media usage

135. Which three TV programmes do you watch most regularly?

1. ………………………………………………… 2. …………………………………………………

3. …………………………………………………

---

136. Which newspapers do you read?

1. ………………………………………………… 2. …………………………………………………

---

137. Which websites do you look at most regularly?

1. ………………………………………………… 2. …………………………………………………

3. ………………………………………………… 4. …………………………………………………

Thrombolysis eligibility (to be filled in by referring to medical notes)

138. Was the patient thrombolysed? ☐ Yes ☐ No

---

139. If not, why not? (tick all that apply)

☐ Too late to give it (more than 4.5 hours at time of CT)

☐ BP more than 185/110

☐ On warfarin

☐ Comorbidity

☐ INR more than 1.3

☐ Brain haemorrhage

☐ Symptoms resolving

☐ Symptoms fully resolved prior to admission

☐ Recent surgery/intervention

☐ Patient declined

Other ____________________

---

140. Would the patient have been a candidate for thrombolysis if they had arrived in less than 4 hours? ☐ Yes ☐ No

---

141. If not, why not? (tick all that apply)

☐ BP more than 185/110

☐ On warfarin

☐ Comorbidity

☐ INR more than 1.3

☐ Brain haemorrhage

☐ Symptoms resolving

☐ Symptoms fully resolved prior to admission

☐ Recent surgery/intervention

Other ____________________

Examination findings (to be filled in from medical notes)

142. Glasgow coma score ____/15

Weakness

143. Face	☐ Yes	☐ No	☐ Right	☐ Left
144. Arm	☐ Yes	☐ No	☐ Right	☐ Left
145. Leg	☐ Yes	☐ No	☐ Right	☐ Left
146. Hand	☐ Yes	☐ No	☐ Right	☐ Left

Sensory Loss

147. Face	☐ Yes	☐ No	☐ Right	☐ Left
148. Arm	☐ Yes	☐ No	☐ Right	☐ Left
149. Leg	☐ Yes	☐ No	☐ Right	☐ Left
150. Hand	☐ Yes	☐ No	☐ Right	☐ Left

Visual Disturbance

151. Hemianopia ☐ Yes ☐ No

152. Gaze palsy ☐ Yes ☐ No

153. Nystagmus ☐ Yes ☐ No

Speech disturbance

154. Dysphasia ☐ Yes ☐ No

155. Dysarthria ☐ Yes ☐ No

Other

156. Ataxia ☐ Yes ☐ No

157. Neglect ☐ Yes ☐ No

158. NIHSS score ____ /30

Cognitive assessment

159. MMSE (from notes) ____ /30

160. AMT (from notes) ____ /10

AMT

161. What is your date of birth? ☐ Correct ☐ Incorrect

---

162. Please repeat and remember this address: 42 West street

---

163. What time is it? ☐ Correct ☐ Incorrect

---

164. What year is it? ☐ Correct ☐ Incorrect

---

165. Where are you? ☐ Correct ☐ Incorrect

---

166. What is my job/job of nurse? ☐ Correct ☐ Incorrect

---

167. What were the years (start and end) of 1st World War? ☐ Correct ☐ Incorrect

---

168. What is the name of the queen? ☐ Correct ☐ Incorrect

---

169. Please count from 20 down to 0 ☐ Correct ☐ Incorrect

---

170. What was that address I gave you to remember earlier? ☐ Correct ☐ Incorrect

---

Total ____ /10

---

171. If no cognitive test done, why not?

……………………………………………………………………………………………………

……………………………………………………………………………………………………

……………………………………………………………………………………………………

Self-rated questionnaire (to be fill in by participant)

Which of the following factors influenced you when you decided to seek **medical** help for your symptoms (i.e. call 999)

---

172. You felt embarrassed to have an ambulance come to the house

☐ Yes ☐ No

---

173. You waited to see if the symptoms would go away without treatment

☐ Yes ☐ No

---

174. You were afraid or mistrustful of doctors, tests, treatments of the diagnosis

☐ Yes ☐ No

---

175. You were worried about the financial cost of being hospitalised (e.g. missing work)

☐ Yes ☐ No

---

176. You did not have access to a telephone

☐ Yes ☐ No

---

177. You were worried about troubling others

☐ Yes ☐ No

178. You did not want to disrupt the activity you were doing at the time of the symptoms

☐ Yes ☐ No

179. You feel that you have no control over your health and that fate will decide what happens

☐ Yes ☐ No

180. You did not think your symptoms were serious

☐ Yes ☐ No

181. Your symptoms were low priority compared with other on-going symptoms you have

☐ Yes ☐ No

182. You had similar symptoms before which got better on their own

☐ Yes ☐ No

183. Your symptoms did not match up to what you thought a stroke was like

☐ Yes ☐ No

184. It did not occur to you that you could be having a stroke/TIA

☐ Yes ☐ No

185. You thought you were having a stroke/TIA but did not think there was anything that could be done to help

☐ Yes ☐ No

186. You thought it would take too long for an ambulance to pick you up so you made your own way to the hospital

☐ Yes ☐ No

187. You thought it would take too long for an ambulance to pick you up so you made your own way to the hospital

☐ Yes ☐ No

---

188. Please mention any other reasons for delay that I have not mentioned

……

……

……

……

Study results

189. Do you wish to receive a summary of the study results by post? ☐ Yes ☐ No

---
